# Supplementary material for: Interferometric Motion Detection in Atomic Layer 2D Nanostructures: Visualizing Signal Transduction Efficiency and Optimization Pathways
Source: Sci Rep. 2016 Jul 28;6:28923. doi: 10.1038/srep28923 (PMC4964573; doi:10.1038/srep28923)
Supplement: Supplementary Information [file srep28923-s1.pdf]

– Supplementary Information –

Interferometric Motion Detection in Atomic Layer 2D Nanostructures: Visualizing Signal Transduction Efficiency and Optimization Pathways

Zenghui Wang, Philip X.-L. Feng*

Department of Electrical Engineering & Computer Science,

Case School of Engineering, Case Western Reserve University,

10900 Euclid Avenue, Cleveland, OH 44106, USA

Table S1. Optimal Geometry for 1L– 200L Graphene, h-BN, and MoS_2 Devices.

2D Material	Graphene			h-BN			MoS_2		
Number of Layers	Crystal Thickness d_1 (nm)	Vacuum Gap d_2 (nm)	Optimal $\|\Re\|$ (%/nm)	Crystal Thickness d_1 (nm)	Vacuum Gap d_2 (nm)	Optimal $\|\Re\|$ (%/nm)	Crystal Thickness d_1 (nm)	Vacuum Gap d_2 (nm)	Optimal $\|\Re\|$ (%/nm)
1	0.335	383	0.051	0.333	351	0.005	0.7	353	0.186
2	0.67	381	0.097	0.666	351	0.010	1.4	350	0.360
3	1.005	378	0.140	0.999	350	0.015	2.1	347	0.515
4	1.34	376	0.179	1.332	350	0.020	2.8	344	0.648
5	1.675	375	0.216	1.665	350	0.025	3.5	342	0.757
6	2.01	373	0.249	1.998	350	0.030	4.2	339	0.844
7	2.345	371	0.280	2.331	350	0.035	4.9	337	0.909
8	2.68	369	0.309	2.664	349	0.040	5.6	335	0.956
9	3.015	368	0.335	2.997	349	0.045	6.3	333	0.988
10	3.35	366	0.360	3.33	349	0.050	7	332	1.007
11	3.685	365	0.383	3.663	349	0.055	7.7	330	1.016
12	4.02	364	0.404	3.996	349	0.060	8.4	329	1.016
13	4.355	363	0.424	4.329	349	0.065	9.1	327	1.011
14	4.69	361	0.442	4.662	348	0.070	9.8	326	1.000
15	5.025	360	0.459	4.995	348	0.075	10.5	325	0.987
16	5.36	359	0.474	5.328	348	0.080	11.2	324	0.970
17	5.695	358	0.489	5.661	348	0.085	11.9	322	0.952
18	6.03	357	0.502	5.994	348	0.090	12.6	321	0.933
19	6.365	356	0.515	6.327	347	0.095	13.3	320	0.914
20	6.7	355	0.527	6.66	347	0.100	14	319	0.894
21	7.035	354	0.537	6.993	347	0.105	14.7	318	0.874

*Corresponding Author. Email: philip.feng@case.edu

**2D Material**	**Graphene**			**h-BN**			**MoS_2**		
Number of Layers	Crystal Thickness d_1 (nm)	Vacuum Gap d_2 (nm)	Optimal $\|\Re\|$ (%/nm)	Crystal Thickness d_1 (nm)	Vacuum Gap d_2 (nm)	Optimal $\|\Re\|$ (%/nm)	Crystal Thickness d_1 (nm)	Vacuum Gap d_2 (nm)	Optimal $\|\Re\|$ (%/nm)
22	7.37	353	0.547	7.326	347	0.110	15.4	318	0.854
23	7.705	353	0.557	7.659	347	0.115	16.1	317	0.835
24	8.04	352	0.565	7.992	346	0.120	16.8	316	0.817
25	8.375	351	0.573	8.325	346	0.125	17.5	315	0.799
26	8.71	350	0.580	8.658	346	0.130	18.2	314	0.782
27	9.045	349	0.587	8.991	346	0.135	18.9	313	0.765
28	9.38	349	0.593	9.324	346	0.140	19.6	312	0.749
29	9.715	348	0.599	9.657	345	0.145	20.3	312	0.734
30	10.05	347	0.604	9.99	345	0.150	21	311	0.720
31	10.385	347	0.608	10.323	345	0.155	21.7	310	0.706
32	10.72	346	0.613	10.656	345	0.160	22.4	309	0.692
33	11.055	345	0.617	10.989	345	0.165	23.1	309	0.680
34	11.39	345	0.620	11.322	344	0.170	23.8	308	0.668
35	11.725	344	0.623	11.655	344	0.175	24.5	307	0.656
36	12.06	344	0.626	11.988	344	0.180	25.2	306	0.645
37	12.395	343	0.629	12.321	344	0.184	25.9	305	0.634
38	12.73	342	0.631	12.654	344	0.189	26.6	305	0.624
39	13.065	342	0.633	12.987	343	0.194	27.3	304	0.614
40	13.4	341	0.635	13.32	343	0.199	28	303	0.604
41	13.735	341	0.636	13.653	343	0.204	28.7	302	0.595
42	14.07	340	0.637	13.986	343	0.209	29.4	301	0.585
43	14.405	340	0.638	14.319	342	0.213	30.1	300	0.576
44	14.74	339	0.639	14.652	342	0.218	30.8	299	0.567
45	15.075	339	0.640	14.985	342	0.223	31.5	299	0.558
46	15.41	338	0.640	15.318	342	0.228	32.2	298	0.548
47	15.745	338	0.640	15.651	342	0.232	32.9	297	0.539
48	16.08	337	0.640	15.984	341	0.237	33.6	296	0.529
49	16.415	337	0.640	16.317	341	0.242	34.3	295	0.519
50	16.75	336	0.640	16.65	341	0.247	35	294	0.509
51	17.085	336	0.640	16.983	341	0.251	35.7	292	0.498
52	17.42	335	0.639	17.316	341	0.256	36.4	291	0.487
53	17.755	335	0.639	17.649	340	0.261	37.1	290	0.476
54	18.09	335	0.638	17.982	340	0.265	37.8	289	0.464
55	18.425	334	0.637	18.315	340	0.270	38.5	287	0.451
56	18.76	334	0.636	18.648	340	0.274	39.2	286	0.437
57	19.095	333	0.635	18.981	339	0.279	39.9	285	0.429
58	19.43	333	0.634	19.314	339	0.284	40.6	283	0.435
59	19.765	332	0.633	19.647	339	0.288	41.3	282	0.440
60	20.1	332	0.631	19.98	339	0.293	42	280	0.446
61	20.435	332	0.630	20.313	339	0.297	42.7	278	0.451
62	20.77	331	0.628	20.646	338	0.302	43.4	276	0.455
63	21.105	331	0.627	20.979	338	0.306	44.1	274	0.458
64	21.44	330	0.625	21.312	338	0.311	44.8	272	0.461

2D Material	Graphene			h-BN			MoS_2		
Number of Layers	Crystal Thickness d_1 (nm)	Vacuum Gap d_2 (nm)	Optimal $\lvert\Re\rvert$ (%/nm)	Crystal Thickness d_1 (nm)	Vacuum Gap d_2 (nm)	Optimal $\lvert\Re\rvert$ (%/nm)	Crystal Thickness d_1 (nm)	Vacuum Gap d_2 (nm)	Optimal $\lvert\Re\rvert$ (%/nm)
65	21.775	330	0.624	21.645	338	0.315	45.5	270	0.463
66	22.11	330	0.622	21.978	337	0.320	46.2	267	0.464
67	22.445	329	0.620	22.311	337	0.324	46.9	264	0.464
68	22.78	329	0.618	22.644	337	0.328	47.6	261	0.462
69	23.115	328	0.616	22.977	337	0.333	48.3	258	0.460
70	23.45	328	0.614	23.31	337	0.337	49	254	0.456
71	23.785	328	0.612	23.643	336	0.341	49.7	250	0.451
72	24.12	327	0.610	23.976	336	0.346	50.4	246	0.444
73	24.455	327	0.608	24.309	336	0.350	51.1	241	0.436
74	24.79	327	0.606	24.642	336	0.354	51.8	235	0.427
75	25.125	326	0.604	24.975	335	0.359	52.5	228	0.417
76	25.46	326	0.601	25.308	335	0.363	53.2	219	0.406
77	25.795	326	0.599	25.641	335	0.367	53.9	392	0.393
78	26.13	325	0.597	25.974	335	0.371	54.6	385	0.380
79	26.465	325	0.595	26.307	335	0.375	55.3	379	0.366
80	26.8	324	0.592	26.64	334	0.380	56	374	0.352
81	27.135	324	0.590	26.973	334	0.384	56.7	370	0.337
82	27.47	324	0.587	27.306	334	0.388	57.4	366	0.321
83	27.805	323	0.585	27.639	334	0.392	58.1	362	0.306
84	28.14	323	0.582	27.972	333	0.396	58.8	359	0.290
85	28.475	323	0.580	28.305	333	0.400	59.5	356	0.275
86	28.81	322	0.577	28.638	333	0.404	60.2	353	0.260
87	29.145	322	0.575	28.971	333	0.408	60.9	350	0.245
88	29.48	322	0.572	29.304	333	0.412	61.6	348	0.230
89	29.815	321	0.569	29.637	332	0.416	62.3	346	0.216
90	30.15	321	0.567	29.97	332	0.420	63	344	0.207
91	30.485	321	0.564	30.303	332	0.424	63.7	342	0.213
92	30.82	320	0.561	30.636	332	0.428	64.4	340	0.219
93	31.155	320	0.558	30.969	331	0.431	65.1	338	0.224
94	31.49	320	0.556	31.302	331	0.435	65.8	336	0.228
95	31.825	319	0.553	31.635	331	0.439	66.5	335	0.231
96	32.16	319	0.550	31.968	331	0.443	67.2	333	0.234
97	32.495	319	0.547	32.301	330	0.447	67.9	332	0.237
98	32.83	318	0.544	32.634	330	0.450	68.6	330	0.238
99	33.165	318	0.541	32.967	330	0.454	69.3	329	0.239
100	33.5	318	0.539	33.3	330	0.458	70	327	0.240
101	33.835	317	0.535	33.633	330	0.461	70.7	326	0.240
102	34.17	317	0.533	33.966	329	0.465	71.4	325	0.240
103	34.505	317	0.530	34.299	329	0.469	72.1	324	0.239
104	34.84	316	0.527	34.632	329	0.472	72.8	323	0.237
105	35.175	316	0.524	34.965	329	0.476	73.5	321	0.236

2D Material	Graphene			h-BN			MoS_2		
Number of Layers	Crystal Thickness d_1 (nm)	Vacuum Gap d_2 (nm)	Optimal $\|\Re\|$ (%/nm)	Crystal Thickness d_1 (nm)	Vacuum Gap d_2 (nm)	Optimal $\|\Re\|$ (%/nm)	Crystal Thickness d_1 (nm)	Vacuum Gap d_2 (nm)	Optimal $\|\Re\|$ (%/nm)
106	35.51	316	0.521	35.298	328	0.479	74.2	320	0.234
107	35.845	316	0.517	35.631	328	0.483	74.9	319	0.232
108	36.18	315	0.514	35.964	328	0.486	75.6	318	0.229
109	36.515	315	0.511	36.297	328	0.490	76.3	317	0.226
110	36.85	315	0.508	36.63	328	0.493	77	316	0.223
111	37.185	314	0.505	36.963	327	0.497	77.7	315	0.220
112	37.52	314	0.502	37.296	327	0.500	78.4	314	0.217
113	37.855	314	0.499	37.629	327	0.503	79.1	313	0.213
114	38.19	313	0.496	37.962	327	0.507	79.8	312	0.210
115	38.525	313	0.492	38.295	326	0.510	80.5	311	0.206
116	38.86	313	0.489	38.628	326	0.513	81.2	310	0.202
117	39.195	312	0.486	38.961	326	0.517	81.9	309	0.198
118	39.53	312	0.483	39.294	326	0.520	82.6	308	0.194
119	39.865	312	0.479	39.627	325	0.523	83.3	307	0.190
120	40.2	311	0.476	39.96	325	0.526	84	306	0.185
121	40.535	311	0.473	40.293	325	0.529	84.7	305	0.181
122	40.87	311	0.469	40.626	325	0.532	85.4	304	0.177
123	41.205	310	0.466	40.959	325	0.536	86.1	303	0.172
124	41.54	310	0.463	41.292	324	0.539	86.8	302	0.167
125	41.875	310	0.459	41.625	324	0.542	87.5	301	0.163
126	42.21	310	0.456	41.958	324	0.545	88.2	300	0.158
127	42.545	309	0.453	42.291	324	0.548	88.9	299	0.154
128	42.88	309	0.449	42.624	323	0.551	89.6	298	0.149
129	43.215	309	0.446	42.957	323	0.554	90.3	296	0.144
130	43.55	308	0.442	43.29	323	0.557	91	295	0.139
131	43.885	308	0.439	43.623	323	0.559	91.7	294	0.135
132	44.22	308	0.435	43.956	323	0.562	92.4	293	0.130
133	44.555	307	0.432	44.289	322	0.565	93.1	292	0.125
134	44.89	307	0.428	44.622	322	0.568	93.8	290	0.120
135	45.225	307	0.425	44.955	322	0.571	94.5	289	0.115
136	45.56	306	0.421	45.288	322	0.573	95.2	288	0.110
137	45.895	306	0.417	45.621	321	0.576	95.9	286	0.108
138	46.23	306	0.414	45.954	321	0.579	96.6	285	0.110
139	46.565	305	0.410	46.287	321	0.582	97.3	283	0.111
140	46.9	305	0.407	46.62	321	0.584	98	282	0.112
141	47.235	305	0.403	46.953	320	0.587	98.7	280	0.114
142	47.57	304	0.399	47.286	320	0.589	99.4	279	0.115
143	47.905	304	0.396	47.619	320	0.592	100.1	277	0.116
144	48.24	304	0.392	47.952	320	0.594	100.8	275	0.117

2D Material	Graphene			h-BN			MoS_2		
Number of Layers	Crystal Thickness d_1 (nm)	Vacuum Gap d_2 (nm)	Optimal $\|\Re\|$ (%/nm)	Crystal Thickness d_1 (nm)	Vacuum Gap d_2 (nm)	Optimal $\|\Re\|$ (%/nm)	Crystal Thickness d_1 (nm)	Vacuum Gap d_2 (nm)	Optimal $\|\Re\|$ (%/nm)
145	48.575	303	0.388	48.285	320	0.597	101.5	273	0.118
146	48.91	303	0.385	48.618	319	0.599	102.2	271	0.118
147	49.245	303	0.381	48.951	319	0.602	102.9	269	0.118
148	49.58	303	0.377	49.284	319	0.604	103.6	266	0.119
149	49.915	302	0.374	49.617	319	0.607	104.3	264	0.118
150	50.25	302	0.370	49.95	318	0.609	105	261	0.118
151	50.585	302	0.366	50.283	318	0.611	105.7	259	0.118
152	50.92	301	0.363	50.616	318	0.614	106.4	256	0.117
153	51.255	301	0.359	50.949	318	0.616	107.1	252	0.116
154	51.59	301	0.355	51.282	317	0.618	107.8	249	0.115
155	51.925	300	0.351	51.615	317	0.621	108.5	245	0.114
156	52.26	300	0.348	51.948	317	0.623	109.2	241	0.112
157	52.595	300	0.344	52.281	317	0.625	109.9	236	0.111
158	52.93	299	0.340	52.614	317	0.627	110.6	230	0.109
159	53.265	299	0.336	52.947	316	0.629	111.3	389	0.107
160	53.6	299	0.333	53.28	316	0.631	112	384	0.105
161	53.935	298	0.329	53.613	316	0.633	112.7	380	0.102
162	54.27	298	0.325	53.946	316	0.635	113.4	376	0.100
163	54.605	298	0.321	54.279	315	0.637	114.1	372	0.097
164	54.94	297	0.318	54.612	315	0.640	114.8	369	0.095
165	55.275	297	0.314	54.945	315	0.641	115.5	366	0.092
166	55.61	297	0.310	55.278	315	0.643	116.2	363	0.089
167	55.945	296	0.306	55.611	314	0.645	116.9	360	0.086
168	56.28	296	0.303	55.944	314	0.647	117.6	358	0.083
169	56.615	296	0.299	56.277	314	0.649	118.3	355	0.080
170	56.95	295	0.295	56.61	314	0.651	119	353	0.077
171	57.285	295	0.292	56.943	314	0.653	119.7	351	0.074
172	57.62	295	0.288	57.276	313	0.654	120.4	349	0.070
173	57.955	294	0.284	57.609	313	0.656	121.1	347	0.067
174	58.29	294	0.281	57.942	313	0.658	121.8	345	0.064
175	58.625	294	0.277	58.275	313	0.660	122.5	343	0.061
176	58.96	293	0.273	58.608	312	0.661	123.2	342	0.058
177	59.295	293	0.270	58.941	312	0.663	123.9	340	0.055
178	59.63	293	0.266	59.274	312	0.665	124.6	339	0.055
179	59.965	292	0.262	59.607	312	0.666	125.3	337	0.056
180	60.3	292	0.259	59.94	311	0.668	126	336	0.057
181	60.635	291	0.255	60.273	311	0.669	126.7	334	0.057
182	60.97	291	0.251	60.606	311	0.671	127.4	333	0.058
183	61.305	291	0.248	60.939	311	0.672	128.1	331	0.059

2D Material	**Graphene**			**h-BN**			**MoS_2**		
Number of Layers	Crystal Thickness d_1 (nm)	Vacuum Gap d_2 (nm)	Optimal $\|\Re\|$ (%/nm)	Crystal Thickness d_1 (nm)	Vacuum Gap d_2 (nm)	Optimal $\|\Re\|$ (%/nm)	Crystal Thickness d_1 (nm)	Vacuum Gap d_2 (nm)	Optimal $\|\Re\|$ (%/nm)
184	61.64	290	0.244	61.272	310	0.674	128.8	330	0.059
185	61.975	290	0.241	61.605	310	0.675	129.5	329	0.060
186	62.31	290	0.237	61.938	310	0.677	130.2	328	0.060
187	62.645	289	0.234	62.271	310	0.678	130.9	326	0.060
188	62.98	289	0.230	62.604	310	0.679	131.6	325	0.061
189	63.315	289	0.227	62.937	309	0.681	132.3	324	0.061
190	63.65	288	0.223	63.27	309	0.682	133	323	0.060
191	63.985	288	0.220	63.603	309	0.683	133.7	322	0.060
192	64.32	288	0.216	63.936	309	0.684	134.4	321	0.060
193	64.655	287	0.213	64.269	308	0.686	135.1	319	0.060
194	64.99	287	0.210	64.602	308	0.687	135.8	318	0.059
195	65.325	286	0.206	64.935	308	0.688	136.5	317	0.059
196	65.66	286	0.203	65.268	308	0.689	137.2	316	0.058
197	65.995	286	0.200	65.601	307	0.690	137.9	315	0.057
198	66.33	285	0.196	65.934	307	0.691	138.6	314	0.057
199	66.665	285	0.193	66.267	307	0.693	139.3	313	0.056
200	67	285	0.190	66.6	307	0.694	140	312	0.055
